# Evaluation of admission levels of P, E and L selectins as predictors for thrombosis in hospitalized COVID-19 patients

**DOI:** 10.1007/s10238-021-00787-9

**Published:** 2022-01-21

**Authors:** Mona M. Watany, Saied Abdou, Reham Elkolaly, Nashwa Elgharbawy, Hossam Hodeib

**Affiliations:** 1 Clinical Pathology Department, Faculty of Medicine, Tanta University, Medical Campus, El Giesh st., Tanta, El-Gharbia Governorate, Tanta, 31527 Egypt; 2 Chest department, Faculty of Medicine, Tanta University, Tanta, Egypt; 3 Internal Medicine Department, Faculty of Medicine, Tanta University, Tanta, Egypt

**Keywords:** SARS-CoV-2, COVID-19, Coagulopathy, P-selectin (CD62P), E-selectin (CD62E), L-selectin (CD62L)

## Abstract

Thromboembolic complications are the most reported cause of death in coronavirus disease-2019 (COVID-19). Hypercoagulability, platelets activation and endotheliopathy are well-recognized features in COVID-19 patients. The aim of this work was to evaluate circulating soluble selectins P, E and L at the time of hospital admission as predictors for upcoming thrombosis. This retrospective study included 103 hospitalized COVID-19 patients and 50 healthy volunteer controls. COVID-19 patients were categorized into two groups; group 1 who developed thrombosis during hospitalization and group 2 who did not. Soluble selectins were quantitated using ELISA technique. Higher levels of sP-selectin, sE-selectin and sL-selectin were detected in COVID-19 patients compared to controls. Furthermore, significantly higher levels were found in group 1 compared to group 2. Their means were [5.86 ± 1.72 ng/mL vs. 2.51 ± 0.81 ng/mL]; [50 ± 8.57 ng/mL vs. 23.96 ± 6.31 ng/mL] and [4.66 ± 0.83 ng/mL vs. 2.95 ± 0.66 ng/mL] for sP-selectin, sE-selectin and sL-selectin respectively. The elevated selectins correlated with the currently used laboratory biomarkers of disease severity. After adjustment of other factors, sP-selectin, sE-selectin and sL-selectin were independent predictors for thrombosis. At sP-selectin ≥ 3.2 ng/mL, sE-selectin ≥ 32.5 ng/mL and sL-selectin ≥ 3.6 ng/mL thrombosis could be predicted with 97.1%, 97.6% and 96.5% sensitivity. A panel of the three selectins provided 100% clinical sensitivity. Admission levels of circulating soluble selectins P, E and L can predict thrombosis in COVID-19 patients and could be used to identify patients who need prophylactic anticoagulants. E-selectin showed a superior clinical performance, as thrombo-inflammation biomarker, to the most commonly studied P-selectin.

## Introduction

Sever acute respiratory syndrome-coronavirus-2 (SARS-CoV-2) is the seventh identified member of coronaviruses family. Four viruses of this family are mostly not pathogenic to humans. SARS‐CoV-1 and MERS‐CoV (Middle East respiratory syndrome-coronavirus) infect humans with mortality rates of more than 10% and more than 35%, respectively [1, 2]. The reported mortality rate in hospitalized patients with coronavirus disease-19 (COVID‐19) caused by SARS-CoV-2 ranges from 4.3 to 15% [3]. More than 70% of patients who do not survive COVID-19 show evidence of thromboembolism [4].

The hypercoagulability observed in COVID‐19 can be explained by three major pathologies that interact very closely and even provide positive feedback loops in between. (A) Coagulopathy: This includes both activation of the extrinsic pathway of coagulation cascade by tissue factor expressed by monocytes in response to IL-6 and IL-1ß, and suppression of fibrinolysis through the increased release of plasminogen activator inhibitor-1 and decreased activity of urokinase plasminogen activator [5]. (B) Platelets activation and increased adhesiveness to leucocytes and damaged endothelium: Platelets are activated directly by the SARS-CoV-2 spike protein and indirectly through mediators released from the damaged endothelia [6]. (C) Endotheliopathy: This is mediated directly through endothelial cell (EC) damage by the infecting virus itself and indirectly through EC activation by cytokines [7].

In view of the need for a rapid response to this sudden unexpected pandemic, drugs already approved for other indications seem to be practically appealing. Identifying circulating biomarkers in COVID-19 patients can help us to understand the pathology behind severe clinical presentation and poor outcome; therefore, providing a rationale to therapeutically try currently available drugs, with already tested safety, used for other diseases that share similar pathologic characters.

Selectins are a family of three closely related glycoproteins; P-selectin, E-selectin and L-selectin. Their corresponding genes reside in tandem on chromosome 1. They act as adhesion receptors that initiate recruitment of leukocytes to the sites of inflammation, tissue injury and immune control [8]. P-selectin (CD62P) is stored in platelets’ *α*-granules and in endothelial cells’ Weibel–Palade bodies and is expressed on their surfaces upon activation [9]. When the extracellular domain is spliced, it is released into circulation and referred to as soluble P-selectin (sP-selectin). E-selectin (CD62E) is produced by ECs upon their activation. Activated ECs release a truncated form of E-selectin, referred to as sE-selectin. Leukocyte L-selectin (CD62L) is expressed on most leucocytes. The ectodomain of L-selectin is rapidly shed from leucocytes’ surface upon activation and circulates as sL-selectin.

This study aimed to evaluate circulating soluble selectins as early thrombo-inflammation markers in COVID-19 patients and to investigate the ability to utilize their blood levels at the time of hospital admission to predict upcoming thrombosis and guide the need for prophylactic anticoagulants. The study may even provide a rationale to try modulating selectins as a therapeutic approach.

## Patients and methods

### Patients’ characteristics and ethical approval

One hundred and three hospitalized patients with confirmed SARS-CoV-2 infection were enrolled in this study. Their cases severity ranged from moderate to severe. Infection was confirmed by positive nasopharyngeal swab testing for COVID-19 by reverse transcriptase-polymerase chain reaction/RT-PCR. Patients were enrolled from November 2020 to the end of April 2021. The study complies with the World Medical Association Declaration of Helsinki and was approved by the local ethical committee, faculty of medicine, Tanta University. Patients approved the utilization of their laboratory test results and their blood samples for research purpose after ensuring their confidentiality. Additionally, fifty healthy volunteers, of matched age and sex, free from cardiovascular diseases, hematological diseases, malignancies, renal and hepatic disorders and free from any infection were recruited as a control group.

The data reported in this study were collected from patients’ admission files and the laboratory results obtained during their hospital stay. Patients were retrospectively divided into two groups according to the development of thrombosis during their hospital stay with D-Dimer level being the indicator. Thrombosis was confirmed by imaging studies when appropriate. Group 1 included 35 patients who developed thrombosis and group 2 included 68 patients who did not develop thrombosis. Patients who had thrombosis at admission, evidenced by elevated D-dimer level > 2.74 nmol/L (> 0.5 mcg/mL) were not included in this study. None of the included patients was under corticosteroid therapy at admission.

### Laboratory analyses

Complete blood count (CBC) was performed using PCE210N cell coulter (ERMA, Tokyo, Japan). D-Dimer was measured using i-chroma™ analyzer (Boditech Med inc., Korea). Il-6 and procalcitonin (PCT) were measured by automated immuno-analyzer, ADVIA centaur^®^ CP (Siemens health care diagnostics Inc., Erlangen, Germany). Lactate dehydrogenase enzyme activity (LDH) and C-reactive protein concentration (CRP) were measured by automated chemistry analyzer, Beckman coulter AU480 (Beckman coulter, California, USA). P, E and L selectin and Von-Willebrand factor (VWF) concentrations were measured by ELISA technique. VWF concentration, expressed as percentage of normal, was measured by VWF total antigen kit (ABP ltd., London, UK). After an extensive search, commercial sandwich ELISA kits from Invitrogen (Invitrogen, Thermo Fisher Scientific Inc., Vienna, Austria) were selected to measure the three selectins because the manufacturers clearly state the absence of interaction between each selectin and the other two types. Soluble P-Selectin was measured using Human sP-selectin ELISA Kit, Catalog Number BMS219-4; with a sensitivity of 0.20 ng/mL and a calculated inter-assay coefficient of variation (CV%) of 5.4%. Soluble E-selectin was measured by Human sE-selectin ELISA Kit, Catalog Number BMS205; with a sensitivity of 0.3 ng/mL and 6% inter-assay CV%. Soluble L-selectin was measured by Human sL-selectin ELISA Kit, Catalog Number BMS206; with a sensitivity of 0.198 ng/mL and an inter-assay CV% of 4.2%. Samples were tested blindly (without prior knowledge to which group they belong). K_3_ EDTA whole blood samples were used for CBC. Citrated plasma was used for the estimation of D-Dimer and VWF concentration. Serum samples were used for the other parameters. All recommended safety procedures were followed during samples collection, handling and waste disposal. For selectins measurement, collected serum samples were stored frozen at − 80 °C till assay time.

### Statistical analysis

The statistical analysis was carried out with SPSS for Microsoft windows, version 20 (IBM, Chicago, USA). Data were checked for normal distribution, the power of the study, and effect size with alpha error set at 0.05. Continuous variables are reported as median and interquartile range (IQR) while categorical variables were presented as frequencies and percentages. Mann–Whitney U test and Chi X^2^ test were used to compare the findings of the two patients’ groups. Kruskal Wallis test was used to compare the medians of the two patients’ groups and control group. Binary logistic regression analysis was used to study selectins’ ability to predict thrombosis in the two COVID patients’ groups. Multinomial regression analysis was used to evaluate the Likelihood to thrombosis in the presence of elevated P, E and L selectins among the three studied groups. Receiver operating characteristic (ROC) curve analysis was used to specify serum levels at which thrombosis could be effectively predicted with best clinical performance (clinical sensitivity and specificity). Also, the correlations between selectins and other laboratory findings were evaluated.

## Results

Considering the multiple laboratory assessments performed as a part of normal practice to follow up hospitalized patients, the laboratory data used for this study are those measured at admission with the exception of D-dimer which was used for grouping the patients into thrombotic and non-thrombotic groups. The duration of hospitalization ranged from 3 to 31 days with a median of 11 days. There was no missing data.

There were no statistically significant differences between the two patients’ groups regarding age, sex, median hospital stays and the incidences of comorbidities. The two groups had significantly different levels of total leukocytes count, lymphocytes percentage, neutrophils/lymphocytes ratio (NLR), platelets count, VWF concentration, D-Dimer, CRP, LDH, IL-6 and PCT. Table 1 summarizes the demographic, clinical and laboratory characteristics of the 103 COVID-19 patients included in the study.

**Table 1:** Demographic, clinical and laboratory characteristics of the studied 103 COVID-19 patients

		Group 1 COVID-19 patients with thrombosis (*N* = 35)	Group 2 COVID-19 patients without thrombosis (*N* = 68)	*Z*	Asymp. Sig
Age [median, (IQR)]		54 (49–60.5)	53.5 (46–61)	− 0.58	0.563
Sex [*n*, (%)]	Male	18 (51.4%)	38 (55.9%)	− 0.43	0.668
	Female	17 (48.6%)	30 (44.1%)		
Hospitalization duration (days) [median, (IQR)]		9 (6–16.5)	11 (8.7–14)	− 1.67	0.095
*Comorbidities [n, (%)]*					
Diabetes		10 (28.6%)	20 (29.4%)	− 0.09	0.929
Hypertension		11 (31.4%)	11 (16.2%)	1.69	0.091
Pre-existing liver disease		5 (14.3%)	6 (8.8%)	0.8	0.425
Pre-existing kidney disease		6 (17.1%)	8 (11.8%)	0.72	0.472
Cardiovascular disease		4 (11.4%)	3 (4.4%)	1.18	0.225†
Autoimmune disease		4 (11.4%)	7 (10.3%)	0.17	1.0†
Malignancy		1 (2.9%)	0		
Pregnancy		2 (5.7%)	0		
TLC (× 10^9^/L) [median, (IQR)]		4.8 ( 3.95–7)	6 (4.3–8.88)	− 1.81	0.072
Lymphocytes % [median, (IQR)])		17 (11–21)	22 (18.8–27.3)	− 3.93	< 0.001*
Neutrophil/lymphocyte ratio [median, (IQR)]		4.4 (3.38–7.36)	3.2 (2.3–3.9)	− 3.93	< 0.001*
Platelets count (× 10^9^/L) [median, (IQR)]		140 (118–210)	181 (135–234)	− 2.33	0.022*
VWF (%) [median, (IQR)]		272 (259–288.5)	238 (230.8–246)	− 6.32	< 0.001*
D-Dimer (nmol/L) [median, (IQR)]^§^		7.98 (5.18–9.93)	0.97 (0.6–1.21)	− 8.29	< 0.001*
CRP (mg/L) [median, (IQR)]		56 (38–71.5)	31.5 (22–43.3)	− 5.12	< 0.001*
LDH (µkat/L) [median, (IQR)]		6.6 (5.28–7.41)	4.24 (3.49–4.98)	− 6.47	< 0.001*
IL-6 (pg/mL) [median, (IQR)]		74 (50–82)	30.5 (22–38)	− 6.46	< 0.001*
PCT (mcg/L) [median, (IQR)]		0.18 (0.09–0.27)	0.13 (0.09–0.18)	− 2.00	0.047*
sP-selectin (ng/mL) [median, (IQR)]		6.31 (4.51–7.37)	2.43 (1.84–3.95)	− 7.67	< 0.001*
sE-selectin (ng/mL) [median, (IQR)]		51 (44–58)	23.5 (19–28)	− 8.17	< 0.001*
sL-selectin (ng/ml) [median, (IQR)]		4.6 (4–5.3)	2.95 (2.4–3.5)	− 7.56	< 0.001*

Patients are divided into two groups according to the development of thrombosis during their hospital stay

Continuous variables are presented as median and interquartile range (IQR); categorical variables are presented as frequencies and percentages. Mann Whitney and Chi *X*^2^ tests are used to compare the findings

*TLC* Total leukocytes count; *VWF* Von Willebrand factor; *CRP* C-Reactive protein; *LDH* lactate dehydrogenase; *IL-6* interleukin-6; *PCT* procalcitonin

*SI unit conversion factors* D-dimer, 1ug/mL = 5.475 nmol/L; CRP, 1 mg/dl = 10 mg/L; LDH, 1U/L = 0.0167 µkat/L

*Indicates significance; † Fisher’s exact test *p* value used due to small samples; § All reported laboratory data were measured at hospital admission except D-Dimer which was measured at the time of thrombosis diagnosis

**Fig. 1:**
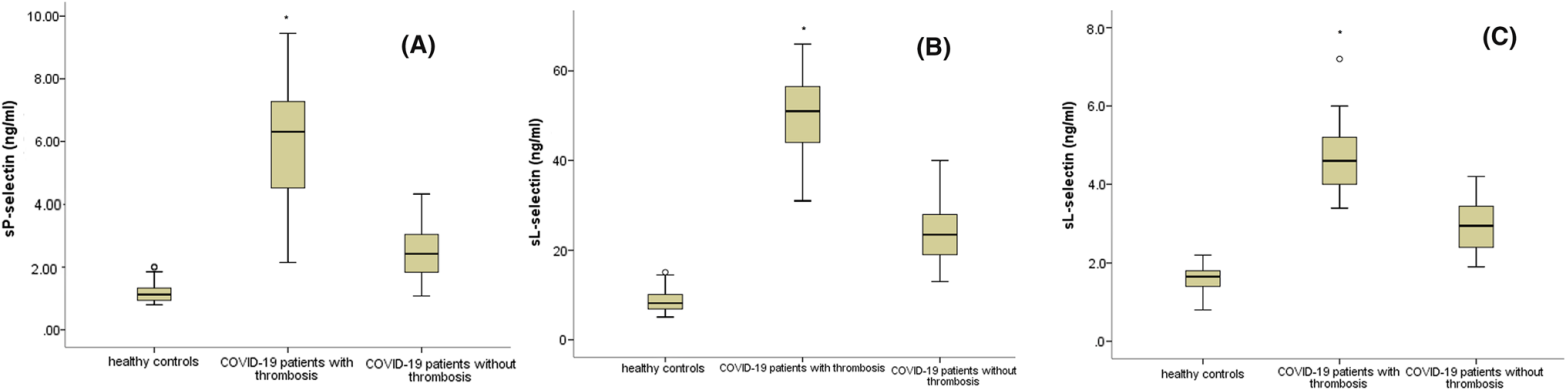
Comparison of P, E and L selectins levels between the three studied groups. *Footnote*: **a** Comparison of sP-selectin level in COVID-19 patients who developed thrombosis to those who did not and to controls. **b** Comparison of sE-selectin level in COVID-19 patients who developed thrombosis to those who did not and to controls. **c** Comparison of sL-selectin level in COVID-19 patients who developed thrombosis to those who did not and to controls. Each box represents the interquartile range, the horizontal line inside the box represent median. Asterisks (*) indicate significant different medians between the three studied groups (Kruskal Wallis test, *p* < 0.05)

sP-selectin level was significantly higher in group 1 (5.86 ± 1.72 ng/mL) compared to group 2 (2.51 ± 0.81 ng/mL) and to controls (1.3 ± 0.33 ng/mL; median 1.13; IQR 0.94–1.34). sE-selectin was significantly higher in group 1 (50 ± 8.57 ng/mL) compared to group 2 (23.96 ± 6.31 ng/mL) and to controls (8.73 ± 2.5 ng/mL, median 8.2; IQR 6.85–10.13). sL-selectin was higher in group 1 (4.66 ± 0.83 ng/mL) compared to group 2 (2.95 ± 0.66 ng/mL) and to controls (1.61 ± 0.31 ng/mL; median 1.65; IQR 1.4–1.8). (Table 1 and Fig. 1).

In summary, there was an overall significant difference between serum soluble selectins P, E and L among the studied groups with evident higher levels (about two folds increase) in COVID-19 patients who developed thrombosis compared to those who did not and compared to healthy controls. All *p* was < 0.001.

Binary regression analysis showed that elevated selectins P, E and L and VWF in COVID-19 patients at the time of hospital admission could predict future thrombosis; in contrast to TLC, NLR, lymphocytes percentage, platelets count, CRP, LDH, IL-6 and PCT that could not (Table 2A). Moreover, multivariate regression showed that thrombosis is likely to develop with elevated selectins (Table 2B).

**Table 2:** Regression analysis test results to evaluate the ability of P, E and L selectins to predict thrombosis in the studied group

(A) Binary logistic regression					
	*B*	S.E	Wald	Sig	Exp(B)
sP-selectin	− 2.234	0.529	17.839	0.023*	0.107
sE-selectin	− 0.403	0.101	16.051	0.011*	0.668
sL-selectin	− 4.195	0.998	17.663	0.016*	0.015
VWF	− 0.077	0.015	26.497	0.012*	0.926
CRP	− 0.066	0.015	19.478	0.105	0.936
TLC	0.036	0.155	0.054	0.816	1.037
lymphocyte	0.021	0.168	0.015	0.901	1.021
NLR	− 0.289	0.577	0.250	0.617	0.749
Platelets	0.002	0.007	0.102	0.749	1.002
LDH	− 0.025	0.007	12.369	0.769	0.976
IL6	− 0.094	0.028	11.324	0.490	0.910
PCT	− 1.433	4.794	0.089	0.758	0.239

(B) multivariate regression (Likelihood Ratio Tests)					
Factor	Effect	Model fitting criteria	Likelihood Ratio Tests		
		− 2 Log likelihood of reduced model	Chi-Square	df	Sig
sP-selectin	Intercept	7.978	0.000	0	
	sP-selectin	314.634	306.656	218	< 0.001*
sE-selectin	Intercept	6.016	0.000	0	
	sP-selectin	318.217	312.201	154	< 0.001*
sL-selectin	Intercept	22.582	0.000	0	
	sP-selectin	278.043	255.462	92	< 0.001*

*TLC* total leukocytes count; *VWF* Von Willebrand factor; *CRP* C-Reactive protein; *NLR* Neutrophils/lymphocytes ratio; *LDH* lactate dehydrogenase; *IL-6* interleukin-6; *PCT* procalcitonin

^*^Statistically significant

sP-selectin showed positive correlations to sE-selectin, sL-selectin, VWF concentration, D-Dimer, CRP, NLR, LDH and IL-6 and a negative correlation to lymphocytes percentage. sE-selectin is positively correlated with sP-selectin, sL-selectin, VWF concentration, D-Dimer, CRP, NLR, LDH and IL-6 and negatively correlated with lymphocytes percentage. sL-selectin had positive correlations to sE-selectin, sP-selectin, VWF concentration, D-Dimer, CRP, NLR, LDH and IL-6 and a negative correlation to lymphocytes percentage. (Table 3).

**Table 3:** Correlation between soluble P-selectin, E-selectin and L-selectin and other laboratory findings

	Selectin-P (ng/ml)		Selectin-E (ng/ml)		Selectin-L (ng/ml)	
	*R*	*P* value	*R*	*P* value	*R*	*P * value
Selectin-E (ng/ml)	0.864	< 0.001*				
Selectin-L (ng/ml)	0.736	< 0.001*	0.721	< 0.001*		
VWF %	0.480	0.014*	0.560	0.002*	0.475	0.062
D-Dimer (ng/ml)	0.795	< 0.001*	0.822	< 0.001*	0.786	< 0.001*
CRP (mg/dl)	0.542	< 0.001*	0.54	< 0.001*	0.532	< 0.001*
TLC (X10^9^/L)	− 0.098	0.213	− 0.179	0.097	− 0.107	0.318
Lymphocyte %	− 0.409	0.042*	− 0.443	0.017*	− 0.329	0.021*
Neutrophis/lymphocytes ratio	0.482	< 0.001*	0.474	0.005*	0.412	0.001*
Platelets count (× 10^9^/L)	− 0.308	0.169	− 0.289	0.246	− 0.287	0.134
LDH (U/L)	0.592	< 0.001*	0.622	< 0.001*	0.533	< 0.001*
IL-6 (pg/ml)	0.675	< 0.001*	0.663	< 0.001*	0.593	< 0.001*
PCT (ng/ml)	0.193	0.758	0.212	0.547	0.165	0.488

*TLC* total leukocytes count; *VWF* Von Willebrand factor; *CRP* C-Reactive protein; *NLR* Neutrophils/lymphocytes ratio; *LDH* lactate dehydrogenase; *IL-6* interleukin-6; *PCT* procalcitonin

^*^Statistically significant

**Fig. 2:**
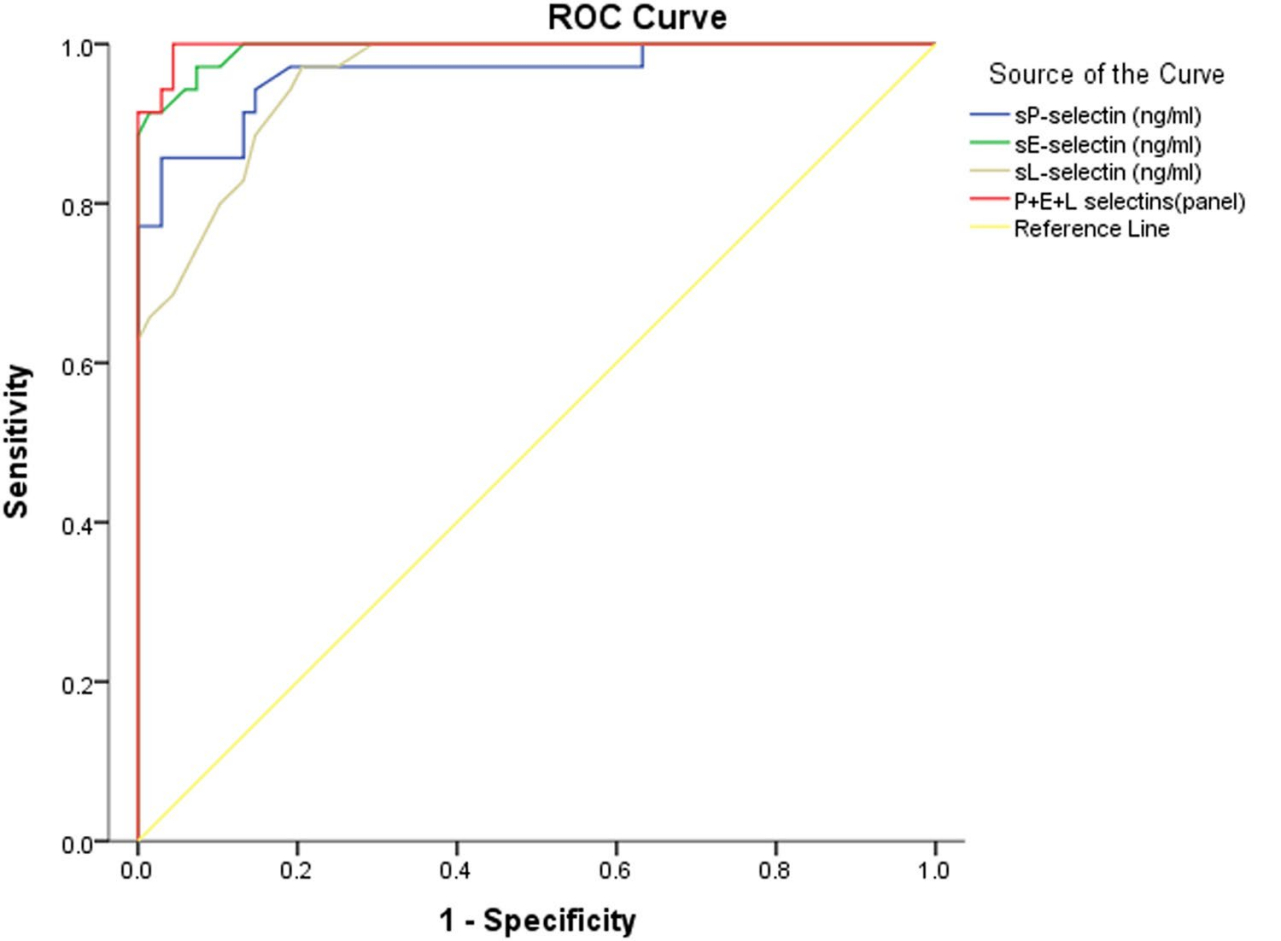
ROC curve analysis of sP-selectin, sE-selectin and sL-selectin as differentiating biomarkers between COVID-19 patients who developed thrombosis and those who did not

Evaluation of the clinical performance of serum sP-selectin, sE-selectin and sL-selectin at admission revealed that they can be used to predict thrombosis with sensitivities of 97.1%, 97.6% and 96.5% respectively; with the best clinical performance achieved with sE-selectin. Additionally, a panel of the three selectins can provide a 100% sensitivity and specificity. Table 4 and Fig. 2 describe the findings of ROC curve analysis.

**Table 4:** Receiver operating characteristic (ROC) curve analyses of soluble P-selectin, E-selectin and L-selectin effectiveness for predicting thrombosis

Test Result Variable(s)	ROC_AUC_ (95% confidence interval)	Cutoff	Sensitivity (%)	Specificity (%)	Std error	Asymptotic sig
sP-selectin (ng/ml)	0.963 (0.923–1)	≥ 3.2	97.1	80.9	0.020	< 0.001*
sE-selectin (ng/ml)	0.993 (0.983–1)	≥ 32.5	98.6	92.6	0.005	< 0.001*
sL-selectin (ng/ml)	0.956 (0.923–0.989)	≥ 3.7	97.1	86.8	0.017	< 0.001*
Combined panel of sP-selectin, sE-selectin and sL-selectin	0.997 (0.991–1)		100	100	0.003	< 0.001*

^*^Statistically significant

## Discussion

The incidence of thromboembolic events is remarkably high in hospitalized patients with moderate and severe COVID-19. The incidence ranges from 20 to 35% [10]. Thrombotic risk even increases in the presence of comorbidities.

Infection with SARS-CoV-2 is associated with release of many inflammatory coagulation mediators which are involved directly in clotting. P-selectin is one of these inflammatory coagulation mediators. In the current study, higher levels of P-selectin were found in COVID-19 patients who developed thrombosis (group 1) compared to those who did not (group 2) and compared to healthy controls. This agrees with Goshua et al. [11] who reported significantly higher levels of markers of ECs and platelets activation, including P-selectin, in COVID-19 patients particularly in patients with worse clinical conditions and poor outcome.

P-selectin is a membrane glycoprotein that mediates the adhesion of activated platelets and ECs to neutrophils and monocytes. This adhesion is responsible for the initial rolling of leukocytes onto the inflamed endothelium, the first step of leukocyte recruitment to inflammation sites. Furthermore, P-selectin activates monocytes to synthesize tissue factor that triggers the extrinsic pathway of blood coagulation [12]. Severe infections, associated with cytokine storms, are associated with Platelet hyper-reactivity. Recently, Bongiovanni et al. [13] proved the over-expression of platelet activation markers, including P-selectin, that relate to the hypercoagulability observed in COVID-19.

P-selectin is expressed during platelets activation and endothelial exocytosis. Both endotheliopathy and platelets activation are crucial for thrombus formation. There are several published data that describe platelet activation signaling pathways where fibrinogen and D-Dimer are involved [14, 15]. MAP kinase and PI3K signaling pathways are switched on within platelets during COVID-19 [16, 17]. Also, SARS-CoV-2 spike protein has a direct stimulatory effect on platelets [6]. Manne et al. [16] reported higher level of P-selectin on the surface of circulating platelets and increased platelets activation from at least some of the COVID-19 patients. Escher et al. [18] also described elevated P-selectin in COVID-19 patients that were directly linked to more severe clinical conditions and to poor prognosis.

In this study sP-selectin positively correlated with D-Dimer, the universally used biomarker for thrombosis. Moreover, elevated sP-selectin at admission proved to be able to predict thrombosis. These findings are in concordance with Bongiovanni et al. and with klok et al. who proved P-selectin over-expression in COVID-19 patients and its relation to the hypercoagulability observed among them [10, 13].

Moreover, in the current study sP-selectin correlated with IL-6, CRP and LDH which are clinically used as indicators of pneumonia severity, cytokine storm evolution and disease progression and are considered poor prognostic factors [19, 20].

Cytokine storm observed in COVID-19 seems to provide a positive feedback loop centered on EC injury. Endothelial exocytosis is a vicious circle; leukocyte and platelet adherence to damaged endothelium lead to microvascular thrombosis and vasculitis. Vasculitis causes tissue injury and releases cytokines like IL-6, IL-1β, and TNFα, which in turn trigger endothelial exocytosis and so on. Thus, SARS-CoV-2 serves as the initiator of a cycle of vascular injury and tissue inflammation which contributes to the hyper-inflammatory hyper-cytokinemia state seen in severe COVID-19 [21].

In this current study, P-selectin positively correlated with VWF. This can be explained by the fact that upon platelets activation, the platelet granules release their content and both P-selectin and VWF are stored in α-granules. Also, ECs store both VWF and P-selectin in the same granule. During EC exocytosis both are released together and interact to promote thrombosis [21].

Statistically significant higher levels of VWF at admission were found in patients who developed thrombosis (group 1) than those who did not (group 2); it was an independent predictor of later thrombosis. VWF has an important role in inflammation, and is considered as one of the acute phase reactants. Reactive oxygen species (ROS) released upon inflammation prompt ECs to release VWF. Moreover, VWF is also a ligand to αvß3 integrin on ECs. This αvß3 integrin has a central role in the endothelial inflammatory responses. Furthermore, VWF binds to two distinct platelet receptors, GPIb of GPIb-IX-V complex and integrin IIb3 of GPIIb-IIIa complex. Binding to those receptors activates intracellular signaling events that lead to full platelet activation and contribute to thrombus formation through binding to fibrinogen. Additionally, intraluminal VWF mediates microvascular occlusion through platelet-independent erythrocyte adhesion to ECs [17].

Our findings generally agree with Grobler et al.-review, where they described increased levels of P-selectin, VWF and D-dimer during COVID-19 progression and recommended that patients should be managed at early stage when there are high levels of fibrinogen, VWF and P-selectin but normal or slightly increased levels of D-dimer. Because as the disease progresses, D-dimer levels rapidly increase, VWF and fibrinogen become depleted and as cytokine storm evolves, higher P-selectin levels can be found. All of these events indicate poor prognosis [17].

Very few studies on E-selectin in COVID-19 have been made. The current study revealed elevated soluble E-selectin (sE-selectin) in COVID-19 patients compared to controls with higher levels observed in patients who developed thrombosis. Furthermore, sE-selectin level correlated with markers of disease severity. This is in concordance with Smadja et al. [22] who analyzed soluble endothelial and angiogenic markers in COVID-19 patients at admission and observed higher levels of E-selectin that could predict disease outcome. Their findings support the role of EC activation and damage in COVID-19 pathogenesis. Similar findings were recently reported in a cohort by Vassiliou et al. [23].

E-selectin (CD62E) is produced solely by ECs, only after their activation by pro-inflammatory mediators like TNFα and IL-1β. E-selectin mediates firm leukocyte adherence to the activated endothelial surface, acts as a chemotactic agent for phagocytic cells, and also shares in angiogenesis. sE-selectin has been in use for years as an early marker of ECs perturbation [24]. Endothelial dysfunction is a major determinant of impaired microcirculatory homeostasis as it shifts the normal vascular tone towards vasoconstriction with subsequent ischemia of organs, induction of inflammation, tissue edema, and the release of tissue factor; thus providing a procoagulant state [25].

Interestingly, in the current study endothelial selectin showed a clinical performance that is superior to P-selectin, the most commonly investigated selectin to date. This adds to the accumulating evidence of the role of EC injury in COVID-19 pathogenesis. SARS-CoV-2 activate endothelial exocytosis either directly through binding to EC surface receptors such as angiotensin-converting enzyme 2 (ACE2) or indirectly through activating host responses and the release of a plethora of cytokines which activate exocytosis. Additionally, SARS-CoV-2 may directly infect ECs and the viral polypeptides themselves activate exocytosis [21].

To our knowledge, this is the first study to investigate the link between soluble L-selectin (sL-selectin) and COVID-19. Higher levels were found in COVID-19 patients compared to healthy controls and higher levels were found in patients who developed thrombosis. L-selectin expression is increased in cytotoxic T-lymphocytes during viral clearance [26]. L-selectin expression can also be increased during neutrophil’s contribution to thrombosis and the formation of neutrophil extracellular traps (NET), a process called immune-thrombosis, which has been described in severe COVID-19 cases [27, 28].

During inflammation, L-selectin mediates secondary capture of leucocytes to the inflammation sites by nucleating leukocyte–leukocyte interactions through binding to P-selectin glycoprotein ligand-1 (PSGL-1) expressed on a vessel wall-adherent monocyte. Leucocytes transiently bind to the adherent monocyte through L-selectin then attach to the activated endothelium downstream from the nucleation site [29].

The elevated levels of sL-selectin correlated with CRP, LDH and IL-6, (i.e., with disease severity). This supports the vicious circle of vasculitis and tissue inflammation described above. In COVID-19 patients, the elevated sL-selectin correlated with lymphopenia. This might be due to consumption of lymphocytes during their adhesion to the activated endothelium; herein, another clue of the role of endotheliopathy, lymphocytes and inflammatory monocytes in micro-thrombi formation.

A positive correlation was found in-between the three selectins. This finding adds to the mounting evidence of the role of a positive feedback loop of coagulopathy, platelets activation and endothelial dysfunction in COVID-19 pathogenesis, which have been described in many review articles [7, 11, 16, 17, 21, 30]. Finally, this positive correlation in between the three selectins P, E and L may provide a rationale to target their common ligand, PSGL-1, or to therapeutically try anti-selectin antibodies or even to temporarily modulate the expression of selectins in COVID-19 patients at risk of thrombosis.

## Conclusions, limitations and recommendations

Serum levels of soluble P-selectin, E-selectin and L-selectin are elevated in moderate and severe cases of SARS-CoV-2 infection. They correlate with disease severity and are independent predictors of emerging thrombosis. The findings of the present study support the interactive role of coagulopathy, platelets and endothelial dysfunction in COVID-19 related thrombosis. The study proved that E-selectin has superior clinical performance to the most commonly reported P-selectin. The authors recommend considering elevated soluble selectins in COVID-19 patients at hospital admission as an indicator for the need of prophylactic anticoagulants. However, the data reported represents a single-center experience and was collected over 6 months only; so further multicenter controlled studies are recommended.

## Abbreviations

SARS COV-2: Sever acute respiratory syndrome-coronavirus 2
COVID-19: Coronavirus disease-2019
EC: Endothelial cell
PSGL-1: P-selectin glycoprotein ligand-1
PCT: Procalcitonin
CRP: C-reactive protein
LDH: Lactate dehydrogenase
NLR: Neutrophil/lymphocyte ratio
VWF: Von Willebrand factor
IL: Interleukin
